# Tilting dependence and anisotropy of anomaly-related magnetoconductance in type-II Weyl semimetals

**DOI:** 10.1038/s41598-019-51846-x

**Published:** 2019-11-06

**Authors:** Hiroaki Ishizuka, Naoto Nagaosa

**Affiliations:** 1 Department of Applied Physics, The University of Tokyo, Bunkyo, Tokyo 113-8656 Japan; 2 RIKEN Center for Emergent Matter Science (CEMS), Wako, Saitama 351-0198 Japan

**Keywords:** Physics, Topological matter

## Abstract

We theoretically study chiral magnetic effect in type-II Weyl semimetals based on a concise formalism for the magnetoconductance in the semiclassical limit. Using the formula, we find that the anomaly-related current is generally dominated by the contribution from the Weyl nodes when the Fermi level is sufficiently close to the nodes. This is related to the fact that the current is proportional to the square of the Berry curvature, which enhances the contribution from the electrons around the Weyl nodes. The increase and the anisotropy of magnetoconductance induced by the tilting is also explained in a comprehensive way.

## Introduction

Weyl semimetals^1–5^ has been studied intensively for its interesting properties and fundamental questions related to Weyl fermions^6^. The Weyl fermions give rise to unique features such as Fermi arcs^5,7,8^, and are reflected in the transport property of materials such as anomalous Hall effect^9–11^ and magnetoconductance (MC)^12,13^. Among them, the MC is studied in relation to chiral anomaly^14^, which results in a magnetic-field-induced current called chiral magnetic effect^15^. These pioneering works considered the high-field limit in which the Landau levels form. On the other hand, a later study pointed out that the chiral anomaly also appears in a weak field limit^16^, in which the chiral anomaly appears as a Berry phase effect. This phenomenon is also studied experimentally after the discovery of Dirac and Weyl semimetals; many candidate materials show a negative magnetoresistance consistent with the theory^12,13,17–20^. These experiments suggests that the unique properties of Weyl electrons are reflected in material properties.

While the Weyl semimetals are considered as a realization of the Weyl fermions, the Weyl electrons in solids is somewhat different from the ideal Weyl Hamiltonian. They typically have tiltings and warpings, neither of which exist in the ideal Weyl Hamiltonian; an extreme case is the type-II Weyl semimetal^21–23^, in which the conduction and valence bands both cross the Fermi level because of a large tilting. Recent studies revealed that these features specific to the Weyl semimetals give rise to rich physical consequences, such as in anomalous Hall effect^24,25^ and nonlinear optical responses^26–34^. The tilting also affect chiral magnetic effect as well. Recent numerical calculation finds a large enhancement of chiral magnetic effect by the tilting^35,36^; they also finds that the chiral magnetic effect is enhanced only when the magnetic field is directed perpendicular to the tilting direction. In addition, a large part of the Fermi surface in type-II Weyl semimetal is not related to the Weyl electrons. Therefore, it is not clear how much of the contriubtion to the transport phenomena comes from the Weyl nodes. However, the effect of the detailed structure of electronic bands on chiral magnetic effect remains to be fully understood.

In this work, we study the general properties of the MC in the weak field limit by introducing a concise general formula which applies to arbitrary model; it is based on Eq. (1). We discuss that this formalism provides an comprehensive understanding on the basic properties of the anomaly-related MC. In particular, we revisit the MC in Weyl Hamiltonian with tilting and a metal with two type-II Weyl nodes^21–23^, of which the anomaly-related current was studied by different methods^22,35,36^. We here show that the anomaly-related current is dominated by the contribution from the Weyl nodes; this implies that the basic properties of the anomaly-related current is understood based on the Weyl Hamiltonian. The tilting dependence of the anomaly-related current is also discussed.

## Results

### Semiclassical theory

A semiclassical theory for the anomaly-related MC^16^ and its extensions^37–39^ were recently proposed. In this work, however, we take a slightly different approach by reformulating the formula for the ${\mathscr{O}}$(*EB*^2^) response^40^ (See Method section for details):

1 $${{\boldsymbol{J}}}_{b}^{\mathrm{(2)}}=-\,{e}^{4}\tau \sum _{n}\,\int \,\frac{d{p}^{3}}{{\mathrm{(2}\pi )}^{3}}[{{\boldsymbol{W}}}_{{\boldsymbol{p}}n}({\boldsymbol{E}}\cdot {{\boldsymbol{W}}}_{{\boldsymbol{p}}n})]({f}_{{\boldsymbol{p}}n}^{0})^{\prime} ,$$

where

2 $${{\boldsymbol{W}}}_{{\boldsymbol{p}}n}\equiv {{\boldsymbol{b}}}_{{\boldsymbol{p}}n}\times ({{\boldsymbol{v}}}_{{\boldsymbol{p}}n}\times {\boldsymbol{B}}),$$

*e* < 0 is the electron charge, *τ* is the relaxation time, and $({f}_{pn}^{0})^{\prime} \equiv -\,\delta (\mu -{\varepsilon }_{pn})$ is the energy derivative of the Fermi-Dirac distribution function at zero temperature (*μ* is the chemical potential and *ε*_***p****n*_ is the energy of the electron with momentum ***p*** and the band index *n*). In Eq. (2), ***v***_***p****n*_ and ***b***_***p****n*_ are the velocity of electrons with momentum ***p*** and band index *n*, respectively.

**Figure 1:**
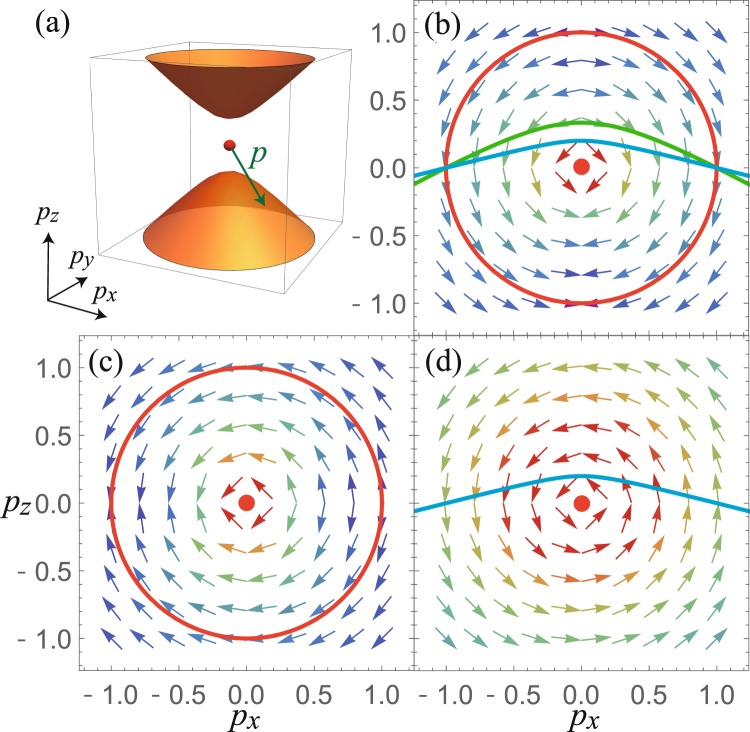
The Fermi surface and ***W***_***p***+_ of type-II Weyl fermion. (**a**) Fermi surface around type-II Weyl node (shown in shaded surfaces). The sphere at the center is the Weyl node and the arrow indicates ***p***. (**b**–**d**) Plot of ***W***_***p***+_ in the *p*_*y*_ = 0 plane. The colors on the arrows reflect the length of ***W***_***p***+_; it is red when ***W***_***p***+_ is large, and blue when small. The red dot at the center is the position of the Weyl node. (**b**) ***W***_***p***+_ with ***B*** = (0, 0, 1). The solid lines are Fermi surfaces with *μ* = 1 and *v*_0_ = 0 (red), 2 (green), and 4 (blue). The same plots for ***B*** = (1, 0, 0) are in (**c**) *v*_0_ = 0 and (**d**) *v*_0_ = 4.

The form of Eq. (1) implies ${(\hat{{\boldsymbol{e}}}\cdot {W}_{{\boldsymbol{p}}n})}^{2} \sim {p}^{-4}$ for the electrons close to a Weyl node [See Fig. 1(a)]. (Here, we assumed the Weyl node is at ***p*** = **0**). Therefore, the contribution to the MC decays rapidly with increasing |***p***|. To make the argument quantitative, we consider a generalized type-II Weyl Hamiltonian $H={R}_{0}({\boldsymbol{p}})+{\sum }_{a=x,y,z}\,{R}_{a}({\boldsymbol{p}}){\sigma }^{a}$ where *R*_0_ and *R*_*a*_ are a power series of *p*_*a*_ and ${\sum }_{a=x,y,z}\,{R}_{a}^{2}({\boldsymbol{p}})=0$ only at ***p*** = **0**; in the below, we call the bands with eigenenergy ${\varepsilon }_{{\boldsymbol{p}}\pm }={R}_{0}\pm \sqrt{{R}_{x}^{2}+{R}_{y}^{2}+{R}_{z}^{2}}$ as ± bands. We further assume that the Fermi surface of this model is given by (*p*, *θ*_±_(*p*, *ϕ*), *ϕ*) where (*p*, *θ*, *ϕ*) is the polar coordinate, and *θ*_±_(*p*, *ϕ*) is a single-valued function that determines the Fermi surface of the ± bands; we assume *θ*_+_(*p*, *ϕ*) > *θ*_−_(*p*, *ϕ*). This essentially assumes the energy monotonically increases along *p*_*z*_, and the two bands has one Fermi surface which extends to *p* → ∞. Then, an integral of a function *F*(***p***) over the Fermi surface reads

3 $$\int \,\frac{d{p}^{3}}{{\mathrm{(2}\pi )}^{3}}{F}_{\pm }({\boldsymbol{p}})\delta ({\varepsilon }_{{\boldsymbol{p}}\pm }-\mu )\propto {\int }_{\lambda }\,dpd\varphi {\frac{p\sin \theta {F}_{\pm }({\boldsymbol{p}})}{|{{\boldsymbol{n}}}_{\theta }\cdot {{\boldsymbol{v}}}_{{\boldsymbol{p}}\pm }|}|}_{\theta ={\theta }_{\pm }(p,\varphi )},$$

where ***n***_*θ*_ is a unit vector along the *θ* axis and *λ* is the ifrared cutoff (it is the shortest distance from the Weyl point to the Fermi surface). Assuming ${\varepsilon }_{{\boldsymbol{p}}\pm }\propto {p}^{\tilde{\eta }}$ and ${F}_{\pm }({\boldsymbol{p}})\propto {p}^{-\tilde{a}}$ at *p* → ∞, the integrand become $\propto {p}^{2-\tilde{a}-\tilde{\eta }}{{g}_{\pm }(\theta ,\varphi )|}_{\theta ={\theta }_{\pm }(p,\varphi )}$, where *g* is a function of *θ* and *ϕ*. Hence, the *p* → ∞ part of the integral in Eq. (3) converges when $\tilde{a}\mathrm{ > 3}-\tilde{\eta }$; this implies that the electrons away from the Weyl nodes does not contribute to the MC. On the other hand, the infrared part of the integral diverges as *λ* → 0 if *a* > 3 − *η* (*ε*_*p*±_ ∝ *p*^*η*^ and *F*_±_(***p***) ∝ *p*^−*a*^ when *p* → 0); the integral remain finite but large, when the Fermi level is slightly away from the node (*λ* is small but not zero).

In case of the ideal type-II Weyl Hamiltonian, *ε*_***p***±_ ∝ *p* and ***b***_***p***±_ ∝ *p*^−2^ for both *p* → 0 and *p* → ∞. Therefore, *F*(***p***) ∝ *p*^−4^ for Eq. (1) which satisfies the above condition 4 = *a* > 3−*η* = 2. This implies that the contribution from the electrons around the Weyl point is dominant. In contrast, a response linearly proportional to ***b***_***p***±_ has *a* = 2 and does not satisfy the above condition. Therefore, the dominant contribution from the Weyl nodes are related to the fact that the chiral magnetic effect is a response in the second-order of the Berry curvature.

In the last, we note that the divergence at *λ* → 0 (which corresponds to the case in which the chemical potential is at the node) is likely to be an artifact of the Boltzmann theory. The Boltzmann theory is valid when the interband scattering is sufficiently small. This assumption holds when the energy difference of two eigenstates at a momentum ***k*** is large. However, the interlayer scattering is important when the difference becomes small, i.e., for the states close to the Weyl node. Assuming the impurity scatterig as the main source of inter-band scattering, the lower limit of *λ* is set by $v\lambda  \sim 1/\tau$, where *v* is the velocity of Weyl cone. Therefore, the above argument is expected to be valid when the distance between the Fermi surface and the Weyl node is larger than 1/(*vτ*). Assuming the relaxation of 10^−13^–10^−12^ s, the lower limit for *vλ* is 10^−1^ meV. Therefore, we expect the above argument is valid for experiments because the doping is usually in the order of 10 meV.

### Type-II Weyl Hamiltonian

We first consider a type-II Weyl Hamiltonian

4 $${H}_{W2}={v}_{\perp }{p}_{x}{\sigma }^{x}+{v}_{\perp }{p}_{y}{\sigma }^{y}+{v}_{z}{p}_{z}{\sigma }^{z}+{v}_{0}{p}_{z}{\sigma }^{0},$$

where *σ*^*a*^ (*a* = *x*, *y*, *z*) is the Pauli matrices and *σ*^0^ ≡ diag(1, 1) is the 2 × 2 unit matrix. By applying Eq. (1), the current along the electric field reads

5 $${J}_{a}=\frac{{\sigma }_{0}{v}_{0}^{3}}{{\mu }^{2}}{f}_{ab}({v}_{z}/{v}_{0},{v}_{\perp }/{v}_{0}){E}_{a}{B}_{b}^{2}$$

with *a*, *b* = *x*, *y*, *z*, where *σ*_0_ = *q*^4^*τ*/(8*π*^2^) is the coefficient for the type-I Weyl node with velocity *v* = 1^16^ and

6a $${f}_{xx}(\alpha ,\beta )=\frac{3{\alpha }^{8}-7{\alpha }^{6}+25{\alpha }^{4}+255{\alpha }^{2}+60}{240{\alpha }^{2}}{\beta }^{2},$$

6b $${f}_{zz}(\alpha ,\beta )=\frac{{\alpha }^{6}-5{\alpha }^{4}+15{\alpha }^{2}+5}{30}{\beta }^{2},$$

when *α* < 1 and

7a $${f}_{xx}(\alpha ,\beta )=\frac{8{\alpha }^{2}+13}{15\alpha }{\beta }^{2},$$

7b $${f}_{zz}(\alpha ,\beta )=\frac{8\alpha }{15}{\beta }^{2},$$

when *α* > 1. The results for *y* is the same as *x*, due to the rotational symmetry about the *z* axis. The chiral magnetic effect also produces transverse magnetoconductivity. They are given by the same form with

8a $${f}_{xz}(\alpha ,\beta )=\frac{-2{\alpha }^{6}+5{\alpha }^{4}+5}{120{\alpha }^{2}}{\beta }^{4},$$

8b $${f}_{zx}(\alpha ,\beta )=\frac{-2{\alpha }^{8}+11{\alpha }^{6}-25{\alpha }^{4}+65{\alpha }^{2}+15}{120{\alpha }^{2}},$$

for *α* < 1 and

9a $${f}_{xz}(\alpha ,\beta )=\frac{{\beta }^{4}}{15\alpha },$$

9b $${f}_{zx}(\alpha ,\beta )=\frac{{\alpha }^{3}+7\alpha }{15},$$

for *α* > 1. These results shoud be valid when the band splitting between the conduction and valence bands on the Fermi surface is larger than the typical interband scattering energy. In the rest of this section, we focus on the longitudinal MC.

In this result, the current along *x* axis is larger than that for the *z* axis when *v*_⊥_ = *v*_*z*_; this trend was discovered in a recent numerical calculation^35^. In our formalism, the anisotropy is understood from the change of ***W***_***p****n*_ [Fig. 1(b–d)]. In the type-II Weyl Hamiltonian, the *z* component of ***v***_***p****n*_ increases with increasing *v*_0_. This change of ***v***_***p****n*_ increases the length of ***W***_***p****n*_ when the magnetic field is perpendicular to the *z* axis [Fig. 1(c,d)], because the length is proportional to ***v***_***p****n*_ × ***B***. We note that ***b***_***p****n*_ does not change by changing *v*_0_. Therefore, the change of ***W***_***p****n*_ by tilting only affects the current induced by *B*_*x*_.

The result also shows both currents increase with increasing *v*_0_; the current along *x* axis increases by ∝*v*_0_^3^ while that for the *z* axis by ∝*v*_0_. This behavior is a consequence of two different reasons: change of the Fermi surface and the change of ***W***_***p****n*_. By increasing *v*_0_, the Fermi surface moves close to the Weyl nodes [Fig. 1(b)]. This gives the divergent increase of the anomaly-related current at *v*_0_ → ∞ for both *x*- and *z*-direction currents. The difference in the power comes from the behavior of ***W***_***p****n*_. As explained in the previous paragraph, ***W***_***p****n*_ for a given ***p*** does not change when the magnetic field is along the *z* axis. On the other hand, it increases linearly with *v*_0_ when the magnetic field is along the *x* axis. As the current is proportional to the square of ***W***_***p****n*_, the power for the *x*-direction current increases by two, which gives ∝ *v*_0_^3^.

### Two Weyl node model

**Figure 2:**
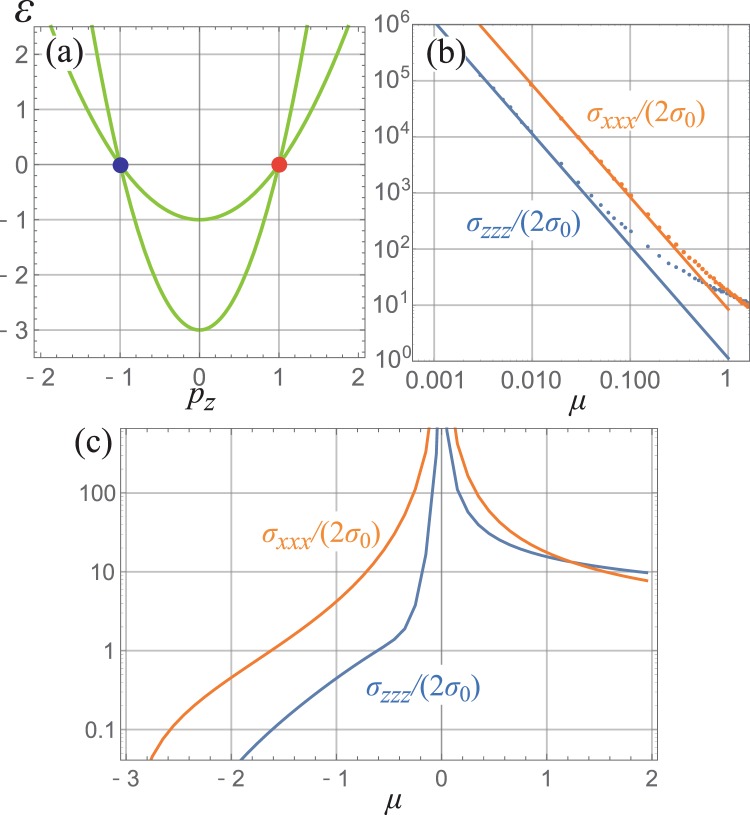
Dispersion and anomaly-related current of the two Weyl node model. (**a**) Dispersion of the Hamiltonian *H*_*D*_ for *m* = 1/4 and *p*_0_ = 1. The two crossings at *p*_*z*_ = ±1 are the Weyl nodes. Nonlinear conductivity for the longitudinal MC (*J*_*b*_^(2)^)^*a*^ = *σ*_*aaa*_*B*_*a*_^2^*E*_*a*_. (**b**) The fitting of the numerical results (dots) using 1/*μ*^2^. The fitted functions are shown by solid lines. All results are for *m* = 1/4 and *p*_0_ = 1. (**c**) Chemical potential *μ* dependence of *σ*_*xxx*_/2*σ*_0_ and *σ*_*zzz*_/2*σ*_0_ calculated numerically.

We next consider a model with two type-II Weyl nodes and investigate whether the anomaly-related current is dominated by the Weyl-node contribution. The Hamiltonian reads:

10 $${H}_{D}={p}_{x}{\sigma }^{x}+{p}_{y}{\sigma }^{y}+({p}_{z}^{2}-{p}_{0}^{2}){\sigma }^{z}+\frac{{p}^{2}-{p}_{0}^{2}}{2m}{\sigma }_{0},$$

where *p*^2^ ≡ *p*_*x*_^2^ + *p*_*y*_^2^ + *p*_*z*_^2^. The band structure of this model along *p*_*x*_ = *p*_*y*_ = 0 line is shown in Fig. 2(a). This model has two Weyl nodes, each located at ***p*** = (0, 0, ±*p*_0_). They are type-I when |*m*| > 1/2 and type-II when |*m*| < 1/2; in the rest, we focus on the case 0 < *m* < 1/2. The band plotted in Fig. 2 is for *m* = 1/4 and *p*_0_ = 1.

The anomaly-related current is calculated numerically using Eq. (1). The nonlinear conductivities for *x*- and *z* directions (*σ*_*xxx*_ and *σ*_*zzz*_, respectively) are shown in Fig. 2(c). Both *σ*_*xxx*_ and *σ*_*zzz*_ shows a divergence at *μ* = 0. The conductivity for *x* is about an order of magnitude larger than that of *z* axis, consistent with the above argument on the type-II Weyl Hamiltonian. Figure 2(b) shows the fitting of *σ*_*aaa*_ (*a* = *x*, *z*) for *μ* > 0 to a function *h*(*μ*) = 2*Cσ*_0_/*μ*^2^, where *C* is a fitting constant. The results fit well with constants *C* = 8.422 and *C* = 1.136 for *σ*_*xxx*_ and *σ*_*zzz*_, respectively; the fitting were done for data in 0 < *μ* < 0.1.

These value of *C* are in good accordance with the analytic results for the Weyl Hamiltonian in Eq. (4). By expanding the model in Eq. (10) around the Weyl point, we find the effective Hamiltonian is Eq. (4) with *v*_⊥_ = 1, *v*_*z*_ = ±2*p*_0_, and *v*_0_ = ±*p*_0_/*m*. Substituting these values into Eq. (6), we obtain ${v}_{0}^{3}{f}_{xx}({v}_{z}/{v}_{0})\simeq 8.348$ and ${v}_{0}^{3}{f}_{zz}({v}_{z}/{v}_{0})\simeq 1.127$, in good agreement with the fitting. The results imply that the anomaly-related MC is dominated by the contribution from the Weyl nodes when *μ* is sufficiently close to the Weyl nodes ($\mu \lesssim 0.1$ in the case of Fig. 2).

### Magnetoconductivity in candidate materials

The above arguments on type-II Weyl Hamiltonian also implies that the estimate of the longitudinal MC ratio may be possible just from the effective Weyl Hamiltonian at the node. We note that the MC ratio is independent of *τ* in the semiclassical limit because both ohmic and the anomaly-related current are linearly proportional to the relaxation time. Therefore, the MC ratio may be estimated without any information about the scattering. Using the Drude formula for the Ohmic current *σ* = *τe*^2^*n*/m^*^ (m^*^ is the effective mass and *n* is the carrier density), the ratio reads

11 $${\chi }_{ab}=\frac{{\sigma }_{ab}}{\sigma }=\frac{{m}^{\ast }{e}^{2}{v}_{0}}{8{\pi }^{2}n\hslash }{f}_{ab}({v}_{\perp }/{v}_{0},{v}_{z}/{v}_{0}){B}_{b}^{2}.$$

Here, we explicitly wrote Planck constant $\hslash$, which was assumed $\hslash$ = 1 in the above sections. The effective Weyl Hamiltonian for WTe_2_ was recently given in ref.^22^, which finds two quartets of Weyl nodes (*W*_1_ and *W*_2_). To make an order estimate, we use *v*_0_ = 2.8 eVÅ, *v*_⊥_ = 0.5 eVÅ, and *v*_*z*_ = 0.2 eV Å for *W*_1_ and *v*_0_ = 1.4 eVÅ, *v*_⊥_ = 0.5 eVÅ, and *v*_*z*_ = 0.2 eV Å for *W*_2_. The carrier density *n* ~ 10^19^ cm^−3^ ^41–43^ and effective mass m^*^ ~ 0.15*m*_*e*_ ^44^, where *m*_*e*_ is the free electron mass is taken from the experiment. Assuming the chemical potential *μ* ~ 10 meV away from the Weyl nodes, we find the largest contribution comes from ${\chi }_{xx} \sim {10}^{-3}{B}^{2}$; this is roughly consistent with recent experiments, which found ~0.1% MC ratio with the magnetic field of order *B* ~ 1 T^45,46^.

Regarding the *μ* dependence, magnetic WSMs^5,19,25^ are a potentially useful setup. Unlike the non-centrosymmetric WSMs, the position (and the existence) of the Weyl nodes can be controlled in a magnetic WSM. In magnetic Weyl semimetals, the position of the Weyl nodes depends on the magnetic configuration such as in EuTiO_3_^25^. EuTiO_3_ hosts four pairs of Weyl nodes when the ferromagnetic moment exists. These Weyl nodes move away from the Γ point with increasing the magnetization; the energy at which the Weyl nodes exist also changes. Therefore, the Weyl nodes go across the Fermi level in the lightly-doped samples where the Fermi level is close to the band bottom at Γ point. This is a potential advantage for studying *μ* dependence, which is achieved by moving the Weyl nodes across the Fermi surface instead of controlling *μ*. Using the model used in ref.^25^ and $\sigma  \sim {10}^{2}$ S/cm, we find ${\chi }_{xx} \sim {10}^{-5}{B}^{2}$; the smaller ratio comes from smaller velocity.

### Linear magnetoconductivity

In a recent work, it was pointed out that the tilting of Weyl cone gives rise to a longitudinal MC which is linearly proportional to the magnetic field^35^. Using the same procedure with Eq. (1), we find the semiclassical formula for linear MC reads

12 $${{\boldsymbol{J}}}_{B}^{\mathrm{(1)}}={e}^{3}\tau \sum _{n=\pm }\,\int \,\frac{d{p}^{3}}{{\mathrm{(2}\pi )}^{3}}{{\boldsymbol{W}}}_{{\boldsymbol{p}}n}({\boldsymbol{E}}\cdot {{\boldsymbol{v}}}_{{\boldsymbol{p}}n})({f}_{{\boldsymbol{p}}n}^{0})^{\prime} -{e}^{3}\tau \sum _{n=\pm }\,\int \,\frac{d{p}^{3}}{{\mathrm{(2}\pi )}^{3}}({\boldsymbol{B}}\cdot {\boldsymbol{E}})({{\boldsymbol{b}}}_{{\boldsymbol{p}}n}\cdot {{\boldsymbol{v}}}_{{\boldsymbol{p}}n}){{\boldsymbol{v}}}_{{\boldsymbol{p}}n}({f}_{{\boldsymbol{p}}n}^{0})^{\prime} \mathrm{.}$$

However, this term vanishes in time-reversal invariant systems. This is shown from the symmetry requirements; *ε*_***p****α*_ = *ε*_*−****p****α*_, ***b***_***p****α*_ = −***b***_−***p****α*_, and ***v***_***p****α*_ = −***v***_−***p****α*_ in the time-reversal invariant systems. This is a manifestation of Onsager’s reciprocal theorem which states *σ*_*aa*_(***B***) = *σ*_*aa*_(−***B***), where *J*_*a*_ = *σ*_*aa*_(***B***)*E*_*a*_; the Weyl Hamiltonian without tilting accidentally possesses the above property of *ε*_***p****α*_, ***b***_***p****α*_, and ***v***_***p****α*_. Therefore, the current in Eq. (12) vanish if no tilting exists. Similarly, the current in Eq. (12) cancels between different nodes in a time-reversal symmetric WSM. Indeed, a recent semiclassical calculation considering time-reversal invariant WSM finds the leading order in MC is proportional to *B*^2^ ^36^. Therefore, the linear MC is a consequene of time-reversal symmetry breaking. Also, as *a* = 2 and *η* = 1, no singular structure is expected from the Weyl nodes. We also note that the absence of *B*-linear current comes from the cancellation between the contribution from ***p*** and −***p***. This is a contrasting feature to Eq. (1), where such a cancellation never occurs. In this work, we focused on the *O*(*EB*^2^) MC because it is the lowest order term that appears regardless of the symmetry.

## Discussion

In this work, we investigated the general properties of the anomaly-related magnetoconductance using the ***W***_***p****n*_ vector formalism in Eq. (1). Focusing on metals with type-II Weyl nodes, we show that the effect of singularity and tilting is intuitively understood by looking at ***W***_***p****n*_ ≡ ***b***_***p****n*_ × (***v***_***p****n*_ × ***B***). In particular, we discussed that the dominant contribution to the magnetoconductance comes from the Weyl nodes; this is because the integrand in Eq. (1) is proportional to the square of a component of ***W***_***p****n*_. On the other hand, the enhancement and the anisotropy of magnetoconductance induced by the tilting is understood from the tilting dependence of ***W***_***p****n*_. We also find that the tilting can enhance the magnetoconductance by more than an order of magnitude.

Unlike the anomaly-related contribution studied here, the normal magnetoconductance due to Lorenz force only depends on the group velocity and the density of states^47^. As neither of these show singularity at the Weyl node, no singular structure is expected for the normal contribution. On the other hand, the singular structure appears for the anomaly-related contribution because it is related to the Berry curvature. Therefore, the observation of chemical potential dependence may provide an experimental evidence for the singular Berry curvature.

The dominant contribution from the Weyl nodes may brings another advantage for studies on materials; it allows estimating the angular dependence of the anomaly-related current only from the effective Weyl Hamiltonian. Usually, magnetoconductance from different mechanisms show different angular dependence. For instance, in the case of the Lorenz force, a positive magnetoconductance appears in the simplest model with symmetric Fermi surface and a perpendicular magnetic field. On the other hand, no magnetoconductivity is seen when the electric and magnetic fields are parallel. Therefore, the different mechanisms are potentially distinguishable from the angular dependence. The dominance of Weyl node contribution is an advantage in this prospect, because the information on the Weyl nodes is sufficient to identify the angular dependence of the magnetoconductance related to the chiral anomaly. Hence, the investigation on the anisotropy is potentially useful for investigating the origin of the magnetoconductance.

Regarding the experiments, our discussion in this work is valid under weak magnetic field with a chemical potential larger than the inverse of the quasi-particle lifetime $\hslash /\tilde{\tau }$. As the semiclassical theory is based on the Boltzmann-type theory, the approximation generally breaks down when the Fermi level is too close to the Weyl nodes; typically, $\mu  > \hslash /\tilde{\tau }$ is required for the validity of the semiclassical approximation. Using $\tilde{\tau } \sim {10}^{-12}$ s, the lower bound for *μ* reads $\hslash /\tilde{\tau } \sim 1$ meV. This is well below the typical doping level $\mu  \sim 10$ meV. Therefore, our theory is valid for experimentally realistic cases.

## Method

### Derivation of Eq. (1)

Equation (1) is obtained from the semiclassical Boltzmann theory^48,49^:

13 $${\partial }_{t}{f}_{{\boldsymbol{p}}n}+\dot{{\boldsymbol{x}}}\cdot {\partial }_{x}{f}_{{\boldsymbol{p}}n}+\dot{{\boldsymbol{p}}}\cdot {\partial }_{{\boldsymbol{p}}}{f}_{{\boldsymbol{p}}n}=-\,\frac{{f}_{{\boldsymbol{p}}n}-{f}_{{\boldsymbol{p}}n}^{0}}{\tau }\mathrm{.}$$

In the right hand side, we used the relaxation-time approximation for the collision integral where the relaxation time is given by *τ*. Here,

14 $$\dot{{\boldsymbol{x}}}={{\boldsymbol{v}}}_{{\boldsymbol{p}}n}+\dot{{\boldsymbol{p}}}\times {{\boldsymbol{b}}}_{{\boldsymbol{p}}n},$$

15 $$\dot{{\boldsymbol{p}}}=e{\boldsymbol{E}}+e\,\dot{{\boldsymbol{x}}}\times {\boldsymbol{B}}\mathrm{.}$$

Assuming the steady state (∂_*t*_ *f*_***p****α*_ = 0) uniform (∂_*x*_ *f*_***p****α*_ = **0**) solution, Eq. (13) becomes

16 $$\dot{{\boldsymbol{p}}}\cdot {\partial }_{{\boldsymbol{p}}}{f}_{{\boldsymbol{p}}n}=-\,\frac{{f}_{{\boldsymbol{p}}n}-{f}_{{\boldsymbol{p}}n}^{0}}{\tau }\mathrm{.}$$

To the linear order in *τ*, the solution of this equation reads

17 $${g}_{{\bf{p}}n}\equiv {f}_{{\bf{p}}n}-{f}_{{\bf{p}}n}^{0},$$

18 $$=-\,\tau {(1+e{\boldsymbol{B}}\cdot {{\boldsymbol{b}}}_{{\bf{p}}n})}^{-1}\times (e{\boldsymbol{E}}+{e}^{2}{{\boldsymbol{v}}}_{{\boldsymbol{p}}n}\times {\boldsymbol{B}}+{e}^{2}({\boldsymbol{E}}\cdot {\boldsymbol{B}}){{\boldsymbol{b}}}_{{\boldsymbol{p}}n})\cdot {{\boldsymbol{v}}}_{{\boldsymbol{p}}n}\,({f}_{{\boldsymbol{p}}n}^{0})^{\prime} ,$$

19 $$\approx -\,\tau \{1-e{\boldsymbol{B}}\cdot {{\boldsymbol{b}}}_{{\boldsymbol{p}}n}+{(e{\boldsymbol{B}}\cdot {{\boldsymbol{b}}}_{{\boldsymbol{p}}n})}^{2}\}\times (e{\boldsymbol{E}}+{e}^{2}{{\boldsymbol{v}}}_{{\boldsymbol{p}}n}\times {\boldsymbol{B}}+{e}^{2}({\boldsymbol{E}}\cdot {\boldsymbol{B}}){{\boldsymbol{b}}}_{{\boldsymbol{p}}n}){{\boldsymbol{v}}}_{{\boldsymbol{p}}n}\,({f}_{{\boldsymbol{p}}n}^{0})^{\prime} ,$$

where $({f}_{p\alpha }^{0})^{\prime} =-\,\delta (\mu -{\varepsilon }_{{k}\alpha })s$ is the energy derivative of the Fermi-Dirac distribution function; here, we focus on the zero-temperature case for simplicity.

The current is obtained by substituting Eq. (19) into the current formula,

20 $${\boldsymbol{J}}=\sum _{n}\,\int \,\frac{d{\boldsymbol{p}}}{{\mathrm{(2}\pi )}^{3}}(1+e{\boldsymbol{B}}\cdot {{\boldsymbol{b}}}_{{\boldsymbol{p}}n})\dot{{\boldsymbol{x}}}\,{f}_{{\boldsymbol{p}}n},$$

21 $$\begin{array}{c}=e\sum _{n}\,\int \frac{d{\boldsymbol{p}}}{{\mathrm{(2}\pi )}^{3}}\{{\boldsymbol{E}}\times {{\boldsymbol{b}}}_{{\boldsymbol{p}}n}+({{\boldsymbol{b}}}_{{\boldsymbol{p}}n}\cdot {{\boldsymbol{v}}}_{{\boldsymbol{p}}n}){\boldsymbol{B}}\}{f}_{{\boldsymbol{p}}n}^{0}\\ \,+\sum _{n}\,\int \,\frac{d{\boldsymbol{p}}}{{\mathrm{(2}\pi )}^{3}}[{{\boldsymbol{v}}}_{{\boldsymbol{p}}n}+e{\boldsymbol{E}}\times {{\boldsymbol{b}}}_{{\boldsymbol{p}}n}+e({{\boldsymbol{b}}}_{{\boldsymbol{p}}n}\cdot {{\boldsymbol{v}}}_{{\boldsymbol{p}}n}){\boldsymbol{B}}]{g}_{{\boldsymbol{p}}n}\mathrm{.}\end{array}$$

The ${\mathscr{O}}$(*EB*^2^) current, ***J***_*b*_^(2)^, appears from the second integral. After some calculation, we find

22 $${{\boldsymbol{J}}}_{b}^{\mathrm{(2)}}=-\,{e}^{4}\tau \sum _{n}\,\int \,\frac{d{p}^{3}}{{\mathrm{(2}\pi )}^{3}}[{{\boldsymbol{W}}}_{{\boldsymbol{p}}n}({\boldsymbol{E}}\cdot {{\boldsymbol{W}}}_{{\boldsymbol{p}}n})]({f}_{{\boldsymbol{p}}n}^{0})^{\prime} ,$$

where

23 $${{\boldsymbol{W}}}_{{\boldsymbol{p}}n}\equiv {{\boldsymbol{b}}}_{{\boldsymbol{p}}n}\times ({{\boldsymbol{v}}}_{{\boldsymbol{p}}n}\times {\boldsymbol{B}}\mathrm{).}$$

In the calculation, we used the identity ***a*** × (***b*** × ***c***) = (***a*** · ***c***)***b***−(***a*** · ***b***)***c***, where ***a***, ***b*** and ***c*** are three-dimensional vectors. Equation (22) is the Eq. (1) in the main text.

The semiclassical formalism has several limitations. First of all, this theory is valid in the weak field limit, where the energy splitting between the Landau levels *ω*_*c*_ are smaller than 1/*τ*. In addition to this general condition, the approximation in Eq. (19) gives an additional constraint; the Maclaurin expansion of 1/(1 + *x*) has a convergence radius of 1. Therefore, *x* < 1 is reqiured, which corresponds to |*e****B*** · ***b***_***p****n*_| < 1 for arbitrary ***p*** on the Fermi surface. However, both conditions have a finite window of *B* where the approximation is justified when the Fermi level is away from the Weyl nodes.
